# Feeding regimens affecting carcass and quality attributes of sheep and goat meat — A comprehensive review

**DOI:** 10.5713/ab.23.0051

**Published:** 2023-06-26

**Authors:** Yafeng Huang, Lumeng Liu, Mengyu Zhao, Xiaoan Zhang, Jiahong Chen, Zijun Zhang, Xiao Cheng, Chunhuan Ren

**Affiliations:** 1 College of Animal Science and Technology, Anhui Agricultural University, Hefei 230036, China; 2 National Agricultural Green Development Long-term Fixed Observation Yingshang Test Station, Yingshang 236200, China; 3 Center of Agriculture Technology Cooperation and Promotion of Dingyuan County, Dingyuan 233200, China

**Keywords:** Carcass Characteristics, Feeding Regimen, Goat, Meat Quality Attributes, Sheep

## Abstract

Sheep and goats can efficiently convert low quality forage into high-quality meat which contains specific nutrients and quality traits. Carcass traits and quality attributes of sheep and goat meat depend upon several factors and one of most effective strategies amongst these is feeding regimens. In this review, the major aspects of feeding regimens affecting growth rate, carcass traits and quality attributes of sheep and goat meat are thoroughly discussed, with a particular focus on physical-chemical composition, flavor profile, and fatty acid (FA) profile. Grazing lambs and kids receiving concentrate or under stall-feeding systems had greater average daily gain and carcass yield compared with animals reared on pasture only. However, growth rate was higher in lambs/kids grazing on pastures of improved quality. Moreover, the meat of grazing lambs receiving concentrate had more intense flavor, intramuscular fat (IMF) content, and unhealthy FA composition, but comparable color, tenderness, juiciness, and protein content compared to that of lambs grazed on grass only. In contrast, meat of concentrate-fed lambs had more intense color, greater tenderness and juiciness, IMF and protein contents, and lower flavor linked to meat. Additionally, the meat of kids grazed on concentrate supplementation had higher color coordinates, tenderness, IMF content and unhealthy FA composition, whereas juiciness and flavor protein content were similar. In contrast, kids with concentrate supplementation had superior color coordinates, juiciness, IMF content and unhealthy FA composition, but lower tenderness and flavor intensity compared to pasture-grazed kids. Thus, indoor-finished or supplemented grazing sheep/goats had higher growth rate and carcass quality, higher IMF content and unhealthy FA composition compared to animals grazed on grass only. Finally, supplementation with concentrate increased flavor intensity in lamb meat, and improved color and tenderness in kid meat, whereas indoor-fed sheep/goats had improved color and juiciness as well as reduced flavor compared to pasture-grazed animals.

## INTRODUCTION

Due to demographic growth, urbanization and changes in consumption behavior, consumers have become increasingly interested in obtaining high-quality and healthy meat originating from sustainable farming practices [1,2]. In this context, sheep and goats are capable of efficiently converting human-inedible-forage into high-value human-edible meat which has a distinguished nutritional composition and sensory quality traits [3]. Moreover, sheep and goat meat is rich in protein with essential amino acids but low intramuscular fat (IMF) content (including cholesterol), and a unique taste [4–6]. In particular, for farmers living in semiarid regions of low- and middle-income countries, sheep and goat meat constitutes nutrition security and livelihood, since rearing beef cattle might be challenging under such conditions [3,7].

It is well known that the growth rate, carcass and quality attributes of sheep and goat meat are influenced by multiple factors in the farming system, i.e., breed/sex, agroclimatic conditions, socio-economic environment, and management system [3,8,9]. In this context, feeding regimens seem to be the most important production factor [10], to a lesser extent season or flock sanitary conditions are also associated to changes in the quantity and nature of feed intake [3]. Previous studies have revealed that the efficiency of feeding regimens to sheep and goat meat can be readily evaluated by carcass weight, degree of conformation and fatness [11,12], which are traits that directly impact farmers’ income, as well as by meat colour and fatty acid (FA) composition [13–15], which in turn affect consumers’ acceptance. Moreover, it has been reported that the feeding regimen implemented by sheep farmers plays a role in determining the quality of the resulting sheep and goat products [3] and affects consumers’ acceptance in terms of tenderness, color and flavor [16]. Compared to concentrate-fed animals, sheep and goats grazing on native pasture had lower growth rate and carcass yield, but higher health benefits as demonstrated by higher contents of total n-3 polyunsaturated fatty acids (PUFA) and lower n-6/n-3 PUFA ratio [10,15,17], combined with specific sensory characteristics preferred by consumers. However, it has been reported that slaughter live weight (SLW) and average daily gain (ADG) of lambs grazing on natural rangeland combined with lucerne (*Medicago arborea*) increased by 5.8% and 18.5%, respectively, accompanied by higher tenderness and meat flavor, which in turn shortened the fattening period compared to indoor-fed animals [18]. In addition, grazing sheep/goats receiving concentrate supplementation had higher carcass weight, and unhealthy FAs composition (e.g., lauric acid and myristic acid) compared to pasture-fed animals [2,19,20]. Therefore, the differences observed in the results of the above-mentioned studies need to be further discussed, and the effects of feeding regimens on sheep and goats require further summarization to provide reliable information for stakeholders in the ruminant production chain, thereby supporting the implementation of effective feeding regimens that will ultimately improve the quality of ruminant-derived products.

In this review, the effects of feeding regimens on growth rate, carcass attributes, and sensory quality of sheep and goat meat are comprehensively discussed. Furthermore, the impact of feeding regimens on chemical composition and FA profiles of sheep and goat meat products are reviewed. The present review aims to serve as the theoretical basis and technical support for improving the efficiency and quality of sheep and goat meat products.

## EFFECT OF SHEEP/GOAT FEEDING REGIMENS ON GROWTH RATE AND CARCASS ATTRIBUTES

The ADG and carcass quality are important parameters for predicting marketable attributes of meat (e.g., meat per animal) [21,22], which directly influences farmers’ income. Feeding regimens can have a marked effect on sheep/goat growth rate and carcass attributes (Table 1). Studies on sheep lambs revealed that the lambs grazing on pasture and supplemented with concentrate or intensive feeding concentrates had higher ADG, SLW, hot carcass weight (HCW), cold carcass weight (CCW) and dressing percentage (DP) compared to animals fed exclusively on native pasture [10,12,15,20,23–26]. These results indicate that supplemented grazing-fed lambs had higher growth performance and carcass quality, since supplemental grazing lambs dedicate higher amount of energy intake, which is corroborated by greater lamb development. However, Devincenzi et al [27] reported that lambs grazing on alfalfa (*Medicago sativa* L.) had similar ADG, SLW and CCW compared to lambs grazing on alfalfa supplemented with barley or concentrate-fed. This observation can be attributed to the fact that barley supplementation does not have advantages over high-quality pastures, and that the higher quality of fresh alfalfa could supply energy supply for lamb growth.

Studies on goat kids have found that ADG, HCW, and DP were higher, whereas SLW was similar or higher in kids grazed on pastures and receiving supplementation compared to animals grazed exclusively on pasture [19,28,29], thus indicating a positive effect of concentrate supplementation on growth rate and meat yield for grazing kids. In addition, compared to grass-fed kids, concentrate-fed kids had higher ADG, heavier SLW, and similar HCW and DP, probably due to the higher energy expenditure required for grazing animals [30], which includes walking and withstanding the hostile environment and weather conditions in pastures (e.g., sunlight, rainfall and dirty) [31]. Furthermore, pasture-fed kids had similar ADG and lower HCW, CCW, and DP compared to concentrate-fed kids [32]. Collectively, these findings can be attributed to forage quality and quantity from pasture as well as to lower gut proportion of concentrate-fed kid, which corroborates the fact that pastures of improved quality did not result in improved carcass characteristics, although they promote growth performance.

Taken together, sheep and goats finished by grazing with supplementation or indoor feeding had higher ADG and superior carcass attributes compared to animals fed exclusively on pastures. In contrast, lambs/kids grazing pastures of improved quality had similar growth rates, although carcass yield of kids did not increase compared to concentrate-fed animals.

## FEEDING REGIMENS AFFECTING SENSORY QUALITY ATTRIBUTES OF SHEEP AND GOAT MEAT

Colour, tenderness, juiciness, and flavor are highly important parameters in the evaluation of meat sensory quality. In particular, meat color plays a significant role in consumer’s quality perception [13,27,33], while variations in tenderness, juiciness and flavor are more likely to influence acceptability and palatability [10,20,22,34].

### Meat colour of *longissimus* muscle

Changes in feeding regimens greatly effects meat colour attributes evaluated in terms of lightness (L*), redness (a*), and yellowness (b*) values (Table 2). Studies on sheep lambs have reported that L*, a* and b* values of *longissimus* muscle of lambs grazed on native pastures and receiving supplementation were similar to those of lambs fed exclusively with pasture [20,27], which indicates that concentrate supplementation did not affect the color of lamb *longissimus* muscle. However, other studies have reported that the *longissimus* muscle of lambs grazing on grassland and receiving concentrate supplementation had higher b* value and comparable a* and b* values than lambs grazing on pasture [25]. In addition, concentrate-fed lambs have higher L* value, and similar or higher a* and b* values compared to lambs fed exclusively on pastures [10,25,34]. These variations can be attributed to greater carcass fatness degree and IMF deposition in concentrate-fed animals because concentrate diets have higher amounts of starch and meat color is influenced by IMF content [35].

Studies on goat kids have described that the *longissimus* muscle of kids raised on grazing pasture had similar L* value and greater a* and b* values compared to kids receiving supplementation on pasture [36], thus indicating that supplementation with concentrate plays a key role in improving goat meat color. This finding could be partly due to carcasses with higher fat content compared to those of animals grazing with supplementation. In addition, Alexandre et al [13] reported higher a* value and similar L* and b* values in the *longissimus* muscle of the indoor-fed kids compared to animals fed exclusively on irrigated pastures, which could be due to the fact that indoor-fed kids had a lower ultimate pH of meat and greater SLW, since a higher pH value of meat result in dark-colored meat, and a higher a* value is related to increased pigmentation related to higher SLW [10].

Overall, supplemented grazing lambs had a similar meat color compared to animals fed exclusively on pastures, while lambs finished with concentrate exhibited superior meat color compared to pasture-fed animals. Finally, the meat of goat kids grazing with supplementation or receiving concentrate had superior meat color compared to that of kids raised exclusively on pasture.

### Tenderness and juiciness of *longissimus* muscle

The Warner-Bratzler shear force (WBSF) is negatively correlated with meat tenderness. Drip loss (DL), water loss (WL), and cooking loss (CL) are negatively correlated with meat juiciness [26,28,34]; whereas water holding capacity (WHC) is positively correlated with muscle juiciness [33]. The feeding regimens influence the meat tenderness and juiciness of sheep lambs and goat kids (Table 3). Studies on sheep lambs have reported that compared to pasture-grazing animals, the *longissimus* muscle of lambs maintained on grazing pastures receiving supplementation with concentrate had similar WBSF and CL [20,25]. Considering that meat WBSF is mainly determined by fiber diameter, connective tissue quantity and IMF content, the above-mentioned observations could be attributed to the fact that, despite the higher IMF content in meat obtained from supplemented grazing lambs, fiber diameter and connective tissue quantity were similar across different feeding regimens. Additionally, the *longissimus* muscle from lambs fed exclusively on pastures had lower less WBSF and CL, but higher WHC compared to that of lambs raised on concentrate diets [10,25,26,34], thus indicating a greater tenderness and juiciness in meat from concentrate-fed animals. These differences can be explained by the fact that indoor-fed lambs have adequate nutrient intake, which in turn promotes IMF deposition, improves marbling, and stimulates the newly synthesized collagen. Moreover, meat tenderness and juiciness are determined by several factors, including intramuscular collagen content, sarcomere length, IMF content and muscle fiber types [25,34,37].

Studies on goat kids have found that the *longissimus* muscle of supplemented grazing kids grazing pasture and receiving concentrate supplementation had similar tenderness (as demonstrated by similar WBSF values) and lower juiciness (as demonstrated by lower CL value) than kids grazing on pasture only [36]. However, Cheng [28] reported the *longissimus* muscle of supplemented grazing kids had higher tenderness and similar juiciness (as indicated by similar DL value) compared with kids grazing on natural grass. These inconsistent results may be related to supplementation levels since higher concentrate levels contribute to improving meat tenderness and juiciness [28]. In contrast, Suo et al [38] evaluated the effect of feeding regimens (grazing pasture vs concentrate finishing) on the meat tenderness and juiciness attributes from Tibetan goat kids and observed lower tenderness and higher juiciness in meat of Tibetan goat kids fed on concentrate (due to higher WBSF and lower WL values) compared to exclusively pasture-raised kids. This could be related to several factors, including diet composition, and required further investigation.

Overall, compared to lambs raised exclusively on pasture, the meat of lambs receiving supplementation had similar tenderness and juiciness, while that of indoor-fed lambs receiving concentrate had greater tenderness and juiciness compared to pasture-finished lambs. In addition, the meat of grazing goat kids receiving high concentrate levels had superior tenderness and similar juiciness compared to those fed exclusively on pastures, while the meat of concentrate-fed kids has lower tenderness and greater juiciness compared to pasture-fed kids.

### Meat flavor of *longissimus* muscle

Different feeding regimens have a significant effect on the flavor of sheep and goat meat (Table 4). Studies on sheep lambs have reported that the *longissimus* muscle of lambs grazed on *Brachiaria* pastures and receiving supplementation at 2.4% body weight (BW) had stronger flavor compared to that of lambs grazing exclusively on pastures [20]). This is likely due to the higher fat tissue content and the different contents of branched-chain FAs in the meat of supplemented grazing lambs. Furthermore, compared to lambs grazing on natural pasture, concentrate-fed lambs had higher 4-methyloctanoic acid (MOA), 4-ethyloctanoic acid (EOA), and 4-methylnonanoic acid (MNA) and lower 4-methylphenol (MP), thus suggesting that the meat from animals in concentrate-finished production system has less appealing flavor [26], since MOA, EOA and MNA content are associated with “mutton” flavor, whereas MP is associated with “pastoral” flavor; “mutton” and “pastoral” flavor are reportedly undesirable odor attributes by consumers [39]. This may be since concentrate diets contain 300% higher crude fat content than that of pasture intake, they promote the synthesis of these volatile compounds in fat tissues. Moreover, compared to lambs grazing on natural rangeland improved by lucerne, indoor-finished lambs exhibited lower intense meat flavor [18], which could be mainly associated with a greater diversity of floristic composition in pasture and increased lucerne intake, thereby resulting in increased deposition of indole and skatole in lamb adipose tissue [40]. However, da Silva et al [20] found that concentrate-fed lambs had higher meat flavor compared to grassland-grazed lambs.

Additionally, studies on goat kids have found that meat from kids fed grazing on pasture and receiving concentrate supplementation had a similar flavor intensity compared to meat from kids grazing on pasture only [36]; this is probably related to the fact that the flavor of cooked goat meat was not affected by supplementation with concentrate. Furthermore, compared to meat from kids grazed exclusively on pasture, meat from indoor-fed kids had lower contents of hexanal, nonanal, 1-pentanol, 1-heptanol, 1-octanol, 2,3-octanedione and higher content of methyl oleate [41], thus corroborating the higher flavor intensity of meat obtained from pasture-grazed goat kids. Yang et al [42] also reported that the *longissimus* muscle of indoor-feeding goats, compared with pasture-grazed goat kids, had less desirable flavor, as indicated by lower isoleucine and leucine contents. These findings can be attributed to the fact that the higher frequency of exercise conducted by pasture-grazed goat accelerates the oxidation rate of mutton fat, thus leading to the production of volatile compounds related to aldehyde and alcohol.

Overall, the meat flavor of supplemented grazing sheep lambs was stronger compared to that of pasture-grazed lambs. In contrast, the flavor of meat obtained from concentrate-finished lambs had lower acceptability compared to that of pasture-grazed lambs. Moreover, the meat of goat kids grazed on pastures and supplemented with concentrate exhibited similar flavor intensity, whereas meat of concentrate-fed goats has less desirable flavor, compared with pasture-grazed lambs.

## FEEDING REGIMENS AFFECTING NUTRITIONAL QUALITY ATTRIBUTES OF SHEEP/GOAT MEAT

### Chemical composition of *longissimus* muscle

Meat nutritional quality attributes, including moisture, protein, IMF, and ash, greatly influence the potential of market consumption as well as consumer acceptability. In this context, protein is a crucial nutrient in meat [43], whereas IMF content is positively correlated with the intensity of “mutton” aroma and flavor attributes [44,45].

It is widely accepted that feeding regimens influence the chemical composition of sheep and goat meat (Table 5). Studies on sheep lambs have found that lambs grazed on native pastures and receiving concentrate diets had a comparable protein content and higher IMF content compared to lambs grazing exclusively on pasture [23,25]. This is mainly due to the higher energy intake of lambs receiving concentrate supplementation. Moreover, compared to lambs fed exclusively on pastures, the *longissimus* muscle of concentrate-fed lambs had a higher IMF content and a similar or higher protein content [10,23,26]. These results could be partly explained by the higher amounts of starch and adequate nutrients in concentrate diets, which contribute to IMF deposition, and increased grain intake resulting in protein degradation and increased protein synthesis in the muscle compared to extensive pasture-fed lambs [46].

Studies on goat kids have described that the *longissimus* muscle of grazing goat kids receiving concentrate or indoor-finished kids had higher IMF content and similar CP content compared to pasture-fed kids [13,28,31]. These differences may result from higher energy intake associated with increased concentrate feeding [47]. From the medical advice point of view, reducing total fat intake can curb the potentially adverse effects of fat intake on the organism, which may include obesity and coronary diseases [48]. Hence, the consumption of sheep and goat meat derived from pasture-based production systems might be preferred as healthy food.

Taken together, lambs and kids grazing on pasture supplemented with concentrate exhibited similar protein content and higher IMF content than animals fed exclusively on pasture. In addition, compared to pasture grazing animals, lambs maintained under intensive feeding regimens had higher IMF content and comparable or higher protein content, while concentrate-fed kids have similar protein content and higher IMF content compared to pasture-fed kids.

### Fatty acid composition of *longissimus* muscle

It is known that the composition of various FAs in meat is correlated with meat quality and human health [49,50]. In this context, saturated FAs (SFA) are often associated with several disorders [51]; in particular, the amount of C12:0, C14:0, and C16:0 may increase the risk of atherosclerosis in humans [52]. In contrast, PUFA, especially n-3 PUFA, linoleic acid (C18:2n6), conjugated linoleic acids (CLAs), α-linolenic acid (ALA, C18:3n3), γ-linolenic acid (C18:3n6), arachidonic acid (C20:4n6), eicosapentaenoic acid (EPA, C20:5n3), docosapentaenoic acid (DPA, C22:5n-3) and docosahexaenoic acid (DHA, C22:6n3) have been regarded as beneficial to human health, helping to prevent the incidence of cardiovascular and anti-inflammatory disorders [53–55], although n-6 PUFA has been closely associated with the incidence of several diseases [56].

Changes in feeding regimens are a major factor affecting the FA profiles of lamb and kid muscle tissue (Tables 6 and 7). Studies on sheep lambs have reported that the *longissimus* muscle of grazing lambs receiving supplementation contains higher content of C16:0 and lower contents of n-3 PUFA, C18:3n6, C18:3n3 and C20:5n3, when compared to lambs grazing on natural grassland [24], which indicates that muscle FA composition of grazing lambs supplemented with concentrate did not improve in terms of health benefits. Gruffat et al [2] also reported that lambs grazing on alfalfa and receiving supplementation have an inferior muscle FA composition, as indicated by lower CLAs content compared to grazed-only lambs. These results could be partly explained by the fact that roughage-rich diets promote the growth of fiber-decomposing microorganisms that conduct hydrogenation in the rumen, which increases the availability of CLA isomers. Additionally, indoor-fed lambs fed receiving concentrate exhibited higher or comparable levels of SFAs (e.g., C12:0, C14:0, and C16:0), and lower or comparable levels of PUFAs (e.g., n-3 PUFA, CLAs, C18:3n6, C18:3n3, C20:5n3, and C22:6n3) compared to lambs grazed exclusively on pastures [2,8,15,24,57], thus indicating that a healthier FA composition in the *longissimus* muscle from lambs reared on a pasture-based fattening system. These differences may be explained by the fact that the higher energy intake by concentrate-fed lambs influenced SFA synthesis, whereas grass is rich in PUFAs such as ALA and C18:2n6, and/or promote ruminal biohydrogenation, thus increasing the production of CLA isomers [24,57,58].

Studies on goat kids have reported that the *longissimus* muscle from kids grazing on pasture supplemented with concentrate had higher levels of C14:0, C16:0, and n-6 PUFA and similar levels of n-3 PUFA compared to kids grazed exclusively on pasture [36]. Another study reported lower levels of n-3 PUFA and linoleic acid but similar levels of C14:0, C16:0 and n-6 PUFA in the meat of supplemented grazing compared to pasture grazing kids [59]. Thus, these results indicate that supplementation with concentrate alters meat FA composition and might negatively impact human nutrition. Moreover, the *longissimus* muscle of concentrate-fed kids had higher levels of C14:0 and C16:0, lower levels of PUFA, n-3 PUFA, C18:2n6, C20:5n3, C22:6n3, and comparable or lower levels of C18:3n3 and C18:3n6 compared to pasture-fed animals [17,38]. The observed differences might be related to the fact that outdoor-fed kids have higher grass intake as well as physical activity, both of which are factors that result in higher n-3 PUFA levels, which also results in meat with a more desirable quality for human consumption, since grass is a natural source of C18:3n3 [24,58]. Yang et al [60] also reported that indoor-fed kids exhibited higher SFA levels, especially C12:0, C14:0, and C16:0, as well as lower nutritive value index, and levels of n-6 PUFA compared with pasture-finished kids, thus suggesting that meat from kids maintained under intensive feeding systems has an unhealthy FA composition compared to that of kids fed exclusively on pastures.

From a health-promoting perspective, the consumption of meat with a lower n-6/n-3 PUFA ratio promotes the suppressive effects of cardiovascular disease and arthritis on cancer [61] and induces arteriosclerosis [62], while a higher PUFA/SFA ratio in meat reduces the risk of developing coronary diseases [63]. It has been reported that the n-6/n-3 PUFA ratio in the *longissimus* muscle from lambs grazed on natural grass was lower than that of meat obtained from lambs receiving supplementation with concentrate [24], which indicates that the FA composition of meat from pasture-fed lambs is healthier. These differences may be related to the fact that providing an increasing amount of concentrate in the diet can reduce n-3 PUFA levels. In contrast to these findings, the n-6/n-3 PUFA ratio was comparable between lambs grazed exclusively on alfalfa and those receiving supplementation with sainfoin [2], which could be attributed to the fact that the level of supplementary feeding did not confer an advantage regarding the PUFA/SFA and n-6/n-3 PUFA ratios. In addition, the n-6/n-3 PUFA ratio in meat obtained from animals submitted to grass feeding regimens was lower than the ratio of n-6/n-3 PUFA in meat originated from stall-fed indoors lambs receiving concentrate diets [2,8,15,42].

Additional studies have described that the *longissimus* muscle from kids grazing pasture supplemented with concentrate had a lower PUFA/SFA ratio and higher n-6/n-3 PUFA ratio compared with kids grazing pasture only [36,59]. In contrast, the *longissimus* muscle of the concentrate-fed kids has a higher n-6/n-3 PUFA ratio, but comparable PUFA/SFA ratio compared to kids exclusively raised on pasture [17,38]. As a result, meat derived from lambs/kids grazing on pasture should be regarded as having a more favorable FA composition for improved health and nutrition.

Overall, compared to pasture grazing systems, supplemental grazing or indoor feeding regimens for lambs/kids resulted in meat with an unhealthy FA composition as demonstrated by higher n-6/n-3 PUFA ratio and lower PUFA/SFA ratio.

## CONCLUSION

In the present review, changes in feeding regimens have an important influence on growth rate, carcass traits and meat quality attributes of sheep and goat meat. Furthermore, it was shown that sheep and goats on a grazing and concentrate supplementation regimen as well as from an indoor feeding regimen had higher growth rates and improved carcass traits (e.g., higher SLW, HCW, CCW, and DP) than animals grazed exclusively on pasture, although grazing on pastures of improved quality promotes lambs/kids growth. Additionally, it was verified that the use of concentrate supplementation enhances flavor intensity of lamb meat as well as improves color and tenderness of kid meat. In contrast, the meat from indoor-finished sheep/goats has improved color and juiciness but reduced flavor intensity compared with animals reared purely on pasture. Finally, meat from indoor-finished or supplemented grazing sheep/goats had higher IMF content and undesired FA composition compared to that of animals reared exclusively on pasture. Future studies are required to elucidate the effects of grazing on moderate or improved pasture types supplemented with proper concentrates as well as of proper grazing time with the moderate concentrate supplementation on growth rate, carcass attributes, and quality of meat derived from small ruminants. Finally, the effect of changes in pasture type and grazing time with moderate concentrate supplementation on sheep and goat meat should also be studied from economic and ecological perspectives.

## Figures and Tables

**Table 1:** Effects of feeding regimens on growth rate and carcass attributes of sheep and goats

Breed	Animals	Animals^1)^/duration (d)	Feeding regimen	Zootechnical indices^2)^	References
Sheep	2-month-old Texel lambs	6/104	Grazing *Brachiaria* pasture, grazing *Brachiaria* pasture supplemented with 2.4% BW, concentrate-fed	↑ SLW, HCW, CCW, DP with supplemented grazing; ↑ SLW, HCW, CCW, DP with concentrate-fed	[20]
	6-month-old male Kheri lambs	20/90	Grazing pasture, grazing pasture supplemented with concentrate, concentrate-fed	↑ ADG, SLW, HCW, DP with supplemented grazing; ↑ ADG, SLW, HCW, DP with concentrate-fed	[23]
	3-month-old male Tan lambs	10/120	Grazing natural grassland, grazing natural grassland supplemented with concentrate	↑ ADG, HCW	[24]
	3-month-old male Tan lambs	10/120	Grazing natural grassland, grazing natural grassland supplemented with concentrate	↑ SLW, DP	[25]
	2-month-old male Romane lambs	12/74	Grazing alfalfa, grazing alfalfa plus supplementation, concentrate-fed	= ADG, SLW, CCW with supplemented grazing; = ADG, SLW, CCW with concentrate-fed	[27]
	5.7-month-old male Norduz lambs	15/84	Grazing natural pasture, concentrate-fed	↑ SLW, HCW, CCW, DP	[10]
	3-month-old Mongolia lambs	10/270	Grazing natural pasture, concentrate-fed	↑ SLW, HCW	[12]
	3-month-old Sunit lambs	10/270	Grazing natural pasture, indoor feeding	↑ SLW, HCW	[15]
	4-month-old Hulunbui female lambs	22/120	Grazing pasture, concentrate-fed	↑ SLW, HCW, DP	[34]
Goats	9-15 month-old Mubende kids	18/104	Grazing grass, grazing grass supplemented with concentrate	= SLW; ↑ ADG, HCW, DP	[19]
	6-month-old Yimeng black kids	25/130	Grazing natural grassland, grazing natural grassland supplemented with concentrate	↑ ADG, SLW, HCW, DP	[28]
	6-month-old castrated crossbred Anglo-Nubian kids	7/n.r.	Grazing irrigated pastures, grazing irrigated grassland plus supplementation	↑ ADG	[29]
	2.9-month-old male Creole kids	60/n.r.	Grazing irrigated pastures, indoor feeding	= HCW, DP; ↑ SLW, ADG	[13]
	3-month-old kids	16/12	Grazing native grass, concentrate-fed	= ADG; ↑ HCW, CCW, DP	[32]
	4-month-old Albas White Cashmere kids	30/60	Grazing natural pasture, concentrate-fed	↑ ADG	[17]

ADG, average daily gain; BW, body weight; CCW, cold carcass weight; DP, dressing percentage; HCW, hot carcass weight; n.r., not reported; SLW, slaughter live weight.

1) Number of animals per each treatment group.

2) = No effect means that no differences were detected among treatments groups (p>0.05); ↑↓ Positive or negative effect, respectively, considering differences when compared to grazing pasture-fed animals (p<0.05).

**Table 2:** Effects of feeding regimens on the color of sheep and goat meat

Breed	Animals	Animals^1)^ /duration (d)	Feeding regimen	Evaluated tissue	Zootechnical indices^2)^	References
Sheep	2-month-old Texel lambs	6/104	Grazing *Brachiaria* pasture, grazing *Brachiaria* pasture supplemented with 2.4% BW	*Longissimus dorsi*	= L*, a*, b*	[20]
	3-month-old male Tan lambs	10/120	Grazing natural grassland, grazing natural grassland supplemented with concentrate, concentrate-fed	*Longissimus thoracis*	= L*, a*; ↑ b* with supplemented grazing; ↑ L*, b*; = a* with concentrate-fed	[25]
	2-month-old male Romane lambs	12/74	Grazing alfalfa, grazing alfalfa plus supplementation	*Longissimus thoracis et lumborum*	= L*, a*, b*	[27]
	5.7-month-old male Norduz lambs	15/84	Grazing natural pasture, concentrate-fed	*Longissimus thoracis*	= a*; ↑ L*, b*	[10]
	4-month-old Hulunbui female lambs	22/120	Grazing pasture, concentrate-fed	*Longissimus dorsi*	= b*; ↑ L*, a*	[34]
Goats	Boer crossbred kids	12/126	Grazing pasture, grazing pasture supplemented with concentrate	*Longissimus dorsi*	= L*; ↑ a*, b*	[36]
	2.9-month-old male Creole kids	60/n.r.	Grazing irrigated pastures, indoor feeding	*Longissimus*	= L*, b*; ↑ a*	[13]

BW, body weight; a*, redness; b*, yellowness; L*, lightness; n.r., not reported.

1) Number of animals per each treatment group.

2) = No effect means that no differences were detected among treatments groups (p>0.05); ↑↓ Positive or negative effect, respectively, considering differences when compared to grazing pasture-fed animals (p<0.05).

**Table 3:** Effects of feeding regimens on the tenderness and juiciness of sheep and goat meat

Breed	Animals	Animals^1)^ / duration (d)	Feeding regimen	Evaluated tissue	Zootechnical indices^2)^	References
Sheep	2-month-old Texel lambs	6/104	Grazing *Brachiaria* pasture, grazing *Brachiaria* pasture supplemented with 2.4% BW	*Longissimus dorsi*	= WBSF, CL	[20]
	3-month-old male Tan lambs	10/120	Grazing natural grassland, grazing natural grassland supplemented with concentrate, concentrate-fed	*Longissimus thoracis*	= CL with supplemented grazing; ↓ CL with concentrate-fed	[25]
	4-month-old male Tibetan lambs	9/120	Grazing desertification grassland, concentrate-fed	*Longissimus lumborum*	= CMP; ↓WBSF, CL, DL	[26]
	4-month-old Hulunbui female lambs	22/120	Grazing pasture, concentrate-fed	*Longissimus dorsi*	= CMP, DL; ↓WBSF; ↑ WHC	[34]
Goats	6-month-old Yimeng black kids	25/130	Grazing natural grassland, grazing natural grassland supplemented with concentrate	*Longissimus dorsi*	= DL, CMP; ↓WBSF	[36]
	Boer crossbred kids	12/126	Grazing pasture, grazing pasture supplemented with concentrate	*Longissimus dorsi*	= WBSF; ↑ CL	[28]
	Newborn Tibetan kids	25/365	Grazing natural grass, concentrate-fed	*Longissimus dorsi*	↓ CMP, WL; ↑ WBSF	[38]

BW, body weight; WBSF, Warner–Bratzler shear force; CL, cooking loss; WHC, water-holding capacity; CMP, cooked meat percentage; DL, drip loss; WL, water loss.

1) Number of animals per each treatment group.

2) = No effect means that no differences were detected among treatments groups (p>0.05); ↑↓ Positive or negative effect, respectively, considering differences when compared to grazing pasture-fed animals (p<0.05).

**Table 4:** Effects of feeding regimens on the flavor of sheep and goat meat.

Breed	Animals	Animals^1)^/duration (d)	Feeding regimen	Evaluated tissue	Zootechnical indices^2)^	References
Sheep	2-month-old Texel lambs	6/104	Grazing *Brachiaria* pasture, grazing *Brachiaria* pasture supplemented with 2.4% BW, concentrate-fed	*Longissimus lumborum*	↑ Flavor with with supplemented grazing; ↑ Flavor with concentrate-fed.	[20]
	6-month-old male Barbarine lambs	6/90	Grazing natural rangeland improved by lucerne, indoor feeding	*Longissimus thoracis*	↓ Flavor	[18]
	4-month-old male Tibetan lambs	9/120	Grazing desertification grassland, concentrate-fed	*Longissimus lumborum*	↑ Benzyl alcohol, 1-heptanol, lcohols; ↓ Decanoic acid, heptanoic acid	[26]
	4-month-old Hulunbui female lambs	22/120	Grazing pasture, concentrate-fed	*Longissimus dorsi*	= MI; ↑EOA, MOA, MNA; ↓ MP	[34]
Goats	Boer crossbred kids	12/126	Grazing pasture, grazing pasture supplemented with concentrate	*Longissimus dorsi*	= Flavor intensity	[36]
	Newborn Wulate kids	6/365	Grazing pasture, indoor feeding	*Longissimus dorsi*	↓ Hexanal, nonanal, 1-pentanol, 1-heptanol, 1-octanol, 2,3-octanedione; ↑ methyl oleate	[41]
	Newborn black kids	3/365	Grazing pasture, indoor feeding	*Longissimus lumborum*	↓ Isoleucine, leucine	[60]

BW, body weight; EOA, 4-ethyloctanoic acid; MOA, 4-methyloctanoic acid; MNA, 4-methylnonanoic acid; MP, 4-methylphenol.

1) Number of animals per each treatment group.

2) = No effect means that no differences were detected among treatments groups (p>0.05), ↑↓ Positive or negative effect, respectively, considering differences when compared to grazing pasture-fed animals (p<0.05).

**Table 5:** Effects of feeding regimens on chemistry composition of sheep and goat meat

Breed	Animals	Animals^1)^ /duration (d)	Feeding regimen	Evaluated tissue	Zootechnical indices^2)^	References
Sheep	6-month-old male Kheri lambs	20/90	Grazing pasture, grazing pasture supplemented with concentrate, concentrate-fed	*Longissimus dorsi*	= protein, ash; ↓ moisture; ↑ IMF with supplemented grazing; = moisture, protein, ash; ↑ IMF with concentrate-fed	[23]
	3-month-old male Tan lambs	10/120	Grazing natural grassland, grazing natural grassland supplemented with concentrate	*Longissimus thoracis*	= moisture, protein, ash; ↑ IMF	[25]
	5.7-month-old male Norduz lambs	15/84	Grazing natural pasture, concentrate-fed	*Longissimus thoracis*	= ash; ↓ moisture; ↑ protein, IMF	[10]
	3-month-old Sunit lambs	10/270	Grazing natural pasture, indoor feeding	*Longissimus dorsi*	= moisture, protein, ash; ↑ IMF	[15]
	4-month-old male Tibetan lambs	9/120	Grazing desertification grassland, concentrate-fed	*Longissimus lumborum*	= moisture, ash; ↑ IMF, protein	[26]
Goats	6-month-old Yimeng black kids	25/130	Grazing natural grassland, grazing natural grassland supplemented with concentrate	*Longissimus dorsi*	= moisture, protein; ↓ ash; ↑ IMF	[28]
	2.9-month-old male Creole kids	60/n.r.	Grazing irrigated pastures, indoor feeding	*Longissimus*	= protein, ash; ↓ moisture; ↑ IMF	[13]
	Newborn Tibetan kids	25/365	Grazing natural grass, concentrate-fed	*Longissimus dorsi*	= protein; ↑ IMF	[38]

IMF, intramuscular fat; n.r., not reported.

1) Number of animals per each treatment group.

2) = No effect means that no differences were detected among treatments groups (p>0.05), ↑↓ Positive or negative effect, respectively, considering differences when compared to grazing pasture-fed animals (p<0.05).

**Table 6:** Effects of feeding regimens on the fatty acid profile of sheep and goat meat

Breed	Animals	Animals^1)^/duration (d)	Feeding regimen	Evaluated tissue	Zootechnical indices^2)^	References
Sheep	5.4-month-old male Romane lambs	12/101	Grazing alfalfa, grazing alfalfa plus supplementation, concentrate-fed	*Longissimus thoracis*	= SFA, C16:0; ↓ C12:0, C14:0 = MUFA; C18:1n9; ↓ C16:1 = PUFA, n-6 PUFA, n-3 PUFA, C18:3n3, C20:5n3, C22:5n3, C22:6 n-3; ↓ CLAs = PUFA/SFA, n-6/n-3 PUFA with supplemented grazing	[2]
					= SFA, C12:0, C14:0, C16:0 = MUFA; C16:1, C18:1n9 = C18:3n6; ↓ PUFA, CLAs, C18:3n3, C20:5n3, C22:5n3, C22:6 n-3 = n-6 PUFA, PUFA/SFA ↓ n-3 PUFA, ↑ n-6/n-3 PUFA with concentrate-fed	
	3-month-old male Tan lambs	10/120	Grazing natural grassland, grazing natural grassland supplemented with concentrate, concentrate-fed	*Longissimus dorsi*	= SFA, C12:0, C14:0; ↑ C16:0 = MUFA, C16:1, C18:1n9 = PUFA, n-6 PUFA, C18:2n6, CLAs, C20:4n6; ↓ n-3 PUFA, C18:3n6, C18:3n3, C20:5n3 ↑ n-6/n-3 PUFA with supplemented grazing	[24]
					= SFA; ↑ C12:0, C14:0, C16:0 = C16:1; ↑ MUFA, C18:1n9 = CLAs, C20:4n6; ↓ PUFA, n-6 PUFA, n-3 PUFA C18:2n6, C18:3n6, C18:3n3, C20:5n3 ↑ n-6/n-3 PUFA with concentrate-fed	
	4-month-old Jezersko–Solčava lambs	8/n.r.	Grazing mountain pasture, concentrate-fed	*Longissimus dorsi*	= C12:0, C14:0; ↑SFA, C16:0 ↑ MUFA, C18:1n9 ↓ PUFA; CLAs, C18:2n6, C18:3n3, C18:3n6, C20:4n6, C20:5n3, C22:5n3, C22:6n3	[57]
	0.5-month-old Ile de France lambs	20/93	Grazing natural pasture-fed, concentrate-fed	*Longissimus dorsi*	↑ SFA, C12:0, C14:0, C16:0 = MUFA, C16:1; ↑ C18:1n9 ↓ PUFA, n-3 PUFA, n-6 PUFA, CLAs, C18:2n6, C18:3n6, C18:3n3, C20:4n6, C20:5n3, C22:5n3, C22:6n3 ↓ PUFA/SFA; ↑ n-6/n-3 PUFA	[8]
	3-month-old Sunit lambs	10/270	Grazing natural pasture, indoor feeding	*Longissimus dorsi*	= SFA, C12:0, C14:0, C16:0 = MUFA, C16:1, C18:1n9 = PUFA; ↓ n-3 PUFA, CLAs, C18:3 n3, C20:5n3, C22:6n3; ↑ n-6 PUFA, C18:2 n6, C20:4 n6 = PUFA/SFA; ↑ n-6/n-3 PUFA	[15]
	4-month-old Tan sheep	12/60	Grazing pasture, indoor feeding	*Longissimus thoracis*	= C12:0, C14:0; ↑ SFA, C16:0; = MUFA; ↑ C16:1, C18:1n9 = C18:3n6; ↓ PUFA, n-3 PUFA, n-6 PUFA, C18:2n6, C18:3n3, C20:4n6, C20:5n3, C22:6n3 ↑n-6/n-3 PUFA	[42]
Goats	Boer crossbred kids	12/126	Grazing pasture, grazing pasture supplemented with concentrate	*Longissimus dorsi*	↑ C14:0, C16:0 ↑ C16:1 = n-3 PUFA; ↑ n-6 PUFA, CLAs ↓ PUFA/SFA; ↑ n-6/n-3 PUFA	[36]
	3-month-old Boer × Kiko (F1) kids	6/79	Grazing pasture, grazing pasture plus supplementation	*Longissimus lumborum*	= SFA, C14:0, C16:0 = MUFA, C16:1, C18:1n9 = PUFA, n-6 PUFA; ↓ n-3 PUFA, C18:2n6 ↑n-6/n-3 PUFA	[59]
	4-month-old Albas White Cashmere goats castrated male kids	30/60	Graze natural pasture, concentrate-fed	*Longissimus thoracis*	= SFA, C12:0; ↑ C14:0, C16:0 ↑ MUFA, C18:1n9 = n-6 PUFA, C20:4n6; ↓ PUFA, n-3 PUFA, C18:2n6, C18:3n3, C18:3n6, C20:5n3, C22:6n3 ↑ n-6/n-3 PUFA	[17]
	Newborn Tibetan kids	25/365	Grazing natural grass, concentrate-fed	*Longissimus dorsi*	= SFA, C12:0; ↑ C14:0, C16:0 = MUFA, C18:1n9 = C18:3n6, C18:3n3; ↓ PUFA = PUFA/SFA	[38]
	Newborn black kids	3/365	Grazing pasture, indoor feeding	*Longissimus lumborum*	↑ SFA, C12:0, C14:0, C16:0 = MUFA, C16:1, C18:1n9 = PUFA, n-3 PUFA, C18:2n6, C18:3n3, C18:3n6, C20:4n6, C20:5n3; ↓ n-6 PUFA	[60]

SFA, saturated fatty acids; MUFA, monounsaturated fatty acids; PUFA, polyunsaturated fatty acids; CLAs, conjugated linoleic acids; n.r., not reported.

1) Number of animals per each treatment group.

2) = No effect means that no differences were detected among treatments groups (p>0.05), ↑↓ Positive or negative effect, respectively, considering differences when compared to grazing pasture-fed animals (p<0.05).

**Table 7:** Summary, mean and standard error of the mean of fatty acids (expressed as mg/100 g of meat) in meat derived from sheep and goat meat reared on different feeding regimens

Fatty acids	Sheep [2,10,14,24–26,57]									Goats [17,38,42,64–66]								
	
	Grass-grazing lambs			Supplemented-grazing lambs			Concentrate-fed lambs			Grass-grazing kids			Supplemented-grazing kids			Concentrate-fed kids		
	
	N	Mean	SEM	N	Mean	SEM	N	Mean	SEM	N	Mean	SEM	N	Mean	SEM	N	Mean	SEM
Total SFA	6	1,060.04	235.62	3	707.36	285.34	7	1,370.59	232.83	2	577.29	44.27	2	1,429.76	398.78	3	1,224.21	294.39
C12:0	4	3.69	1.25	3	1.99	0.83	5	4.25	1.29	2	0.69	0.20	2	25.54	7.73	3	12.75	9.22
C14:0	5	86.41	36.89	3	31.23	13.35	6	80.98	23.56	2	19.96	1.54	2	63.71	19.03	3	56.09	13.48
C16:0	5	473.16	137.22	3	326.23	127.23	6	606.08	121.98	2	275.88	3.70	2	473.81	135.25	3	614.20	51.70
C18:0	4	315.05	84.14	3	337.45	149.64	5	475.87	110.95	2	228.69	47.31	2	437.79	54.83	3	339.35	69.53
C20:0	2	3.87	0.50	2	1.72	1.22	3	3.09	1.27	2	0.47	0.05	2	44.02	13.95	3	21.69	17.21
Total MUFA	5	755.38	175.53	3	611.31	234.85	6	1,103.04	203.92	2	600.21	124.97	2	993.42	343.21	3	1,143.90	253.79
C14:1	4	3.63	1.51	2	0.06	0.02	4	6.86	2.95	2	2.47	0.70	2	16.54	5.96	3	10.84	6.06
C16:1	5	36.24	9.52	3	19.11	7.44	6	44.55	9.17	2	17.20	10.45	2	44.87	12.26	3	29.03	14.17
C18:1n-9	5	668.06	160.21	3	534.18	207.55	6	991.35	183.27	2	555.45	128.15	2	834.86	287.24	3	1,091.62	180.15
Total PUFA	5	307.81	86.90	3	206.28	88.94	6	322.47	67.01	2	29.59	9.08	2	403.08	111.14	3	210.07	139.03
C18:2n6	5	99.46	26.89	3	90.84	33.71	6	154.01	31.30	2	21.62	3.76	2	118.08	32.99	3	72.50	38.43
C18:3n3	4	41.37	14.75	3	18.29	11.35	5	44.96	18.32	2	0.67	0.48	2	31.57	11.07	3	17.92	12.70
C18:3n6	4	1.67	0.57	3	2.25	1.33	5	7.15	3.62	2	0.49	0.04	2	18.78	5.73	3	10.13	6.92
C20:4n6	3	39.75	16.23	3	30.82	11.57	4	47.07	13.58	1	0.00		2	79.76	21.80	2	53.90	37.66
C20:5n3	3	21.66	8.50	3	12.65	9.09	4	14.68	6.78	1	0.00		2	32.05	8.56	2	22.80	15.85
CLAs	3	17.90	7.23	2	9.97	6.81	3	11.95	5.70	1	10.64					1	15.56	
C22:5 n-3	2	36.32	2.32	2	19.80	14.00	3	20.40	9.52									
C22:6 n-3	2	13.60	2.05	2	8.40	5.94	3	7.33	3.59				2	28.95	8.86	1	42.18	0.00
n-3 PUFA	3	100.81	40.14	3	50.63	36.37	4	78.55	42.65	1	0.00		2	116.59	34.04	2	86.33	60.14
n-6 PUFA	3	188.25	72.51	3	143.53	53.26	4	219.41	61.75	1	7.13		2	292.97	83.38	2	195.70	135.96
PUFA/SFA	5	0.35	0.05	3	0.38	0.04	6	0.26	0.02	2	0.21	0.13	3	0.35	0.03	4	0.28	0.08
n-6/n-3 PUFA	4	2.85	0.53	3	3.90	0.97	5	7.83	2.88	1	6.43		2	2.51	0.02	2	5.32	2.01

N, number of supporting reports; SEM, standard error of the mean; SFA, saturated fatty acids; MUFA, monounsaturated fatty acids; PUFA, polyunsaturated fatty acids.
